# Jung on the Nature and Interpretation of Dreams: A Developmental Delineation with Cognitive Neuroscientific Responses

**DOI:** 10.3390/bs3040662

**Published:** 2013-11-22

**Authors:** Caifang Zhu

**Affiliations:** Institute of Analytical Psychology, Department of Applied Psychology, City University of Macau, Avenida Xian Xing Hai, Ed. Golden Dragon Centre, 19° andar, Macau; E-Mail: caifangzhu@hotmail.com; Tel.: +853-2878-1698; Fax: +853-2878-1691

**Keywords:** dreams, Jung, compensation, imagistic and organismic–holistic cognition, cognitive neuroscience, activation–synthesis theory

## Abstract

Post-Jungians tend to identify Jung’s dream theory with the concept of compensation; they tend to believe that Jung’s radically open stand constitutes his dream theory in its entirety. However, Jung’s theory regarding dreams was a product of an evolving process throughout his whole intellectual and professional life. Unfortunately, the theory has not been understood in such a developmental light. Based on a historical and textual study of all dream articles found throughout *The Collected Works of C.G. Jung*, this paper maps a concise three-phase trajectory of Jung’s changing views on dreams and interpretation. The paper posits that Jung’s last essay, “Symbols and the Interpretation of Dreams” (1961), epitomizes his final stand, although such a stand is also reflected in a less explicit and less emphatic way during the latter period of the second phase. The paper also briefly addresses where Jung and Jungians have been enigmatic or negligent. For example, it has not been explicated fully why compensation as slight modifications and compensation as parallels to waking life situations are rare in Jung’s cases In addition, contemporary cognitive and neuroscientific approaches to the study of dreams, as represented by Harry Hunt, William Domhoff, and Allan Hobson, among others, are presented in connection with Jung. The juxtaposition of Jungian, cognitive, and neuroscientific approaches showcases how cognitive and scientific findings challenge, enrich, and in some ways confirm Jung’s dream theory and praxis.

## 1. Introduction

My study of Jung on dreams extracted all the articles that are in *The Collected Works of C.G. Jung* (*CW* hereafter). I performed a comprehensive search of all essays explicitly citing the word “dream” in the title (although there is no doubt that Jung’s references and discussions on dreams are spread much farther and wider throughout numerous other writings in *CW*). The *General Index to CW* provides a long classified index (See [1], pp. 220–232) to virtually every noticeable piece of information pertinent to Jung’s writings on dreams. Eleven articles, including one foreword, are scattered in 6 of the 20 volumes of *CW*, listed in ascending order by volume number as follows:

Vol. 2: Association, Dream and Hysterical Symptom (1904/1910)
Vol. 4: The Analysis of Dreams (1909)
  On the Significance of Number Dreams (1910)
  Morton Prince, “Mechanism and Interpretation of Dreams”: A Critical Review (1911)
Vol. 8: General Aspects of Dream Psychology (1916/1948)
  On the Nature of Dreams (1945/1948)
Vol. 12: Individual Dream Symbolism in Relation to Alchemy (1936)
Vol. 16: The Practical Use of Dream-Analysis (1934)
Vol. 18: Symbols and the Interpretation of Dreams (1961)
  Sigmund Freud: On Dreams (1901)
  Foreword to Fierz-David: *The Dream of Poliphilo* (1946)

Among these articles, the following three are presumably the most representative: “Association, Dream and Hysterical Symptom,” “On the Nature of Dreams,” and “Symbols and the Interpretation of Dreams.” Each of these works epitomizes one of the three major developmental phases of Jung’s theory and practice of dream interpretation. “Association, Dream and Hysterical Symptom” [2] highlights the first phase of Jungian theory. In this phase, Jung was a staunch supporter and advocate of Freud’s repression and sex-drive-based theory of dream interpretation. “On the Nature of Dreams” [3], which is supplemented by “General Aspects of Dream Psychology” [4], as well as by “The Practical Use of Dream-Analysis” [5], marks the second phase. Here, we see the maturation of Jung’s own theory of dreams as compensatory catharsis for one’s waking life. This transformation in Jung’s theoretical position spans a long period between 1916, the year the first version of “General Aspects of Dream Psychology” was published, and the late 1950s. Jung’s work spanning the third or last phase, as represented by “Symbols and the Interpretation of Dreams” [6], illustrates an open, fluid, and pragmatic attitude towards the compensation theory developed during the second phase. In the third phase, Jung concludes that compensation theory is just a proposed hypothesis, however promising it might appear, which can be built upon and further scientifically explored. 

## 2. Solidarity with Freud

The tone of Jung’s partisanship with, and commitment to, Freud is somewhat mitigated in Jung’s autobiography, entitled *Memories, Dreams and Reflections (MDR)* [7]. Jung’s interest in Freud’s theory is more strongly expressed in his early essays on dreams. Jung’s earliest published dream article entitled, “Sigmund Freud: On Dreams” [8], is basically a summary of Freud’s seminal work, *The Interpretation of Dreams* [9]. Jung recapitulates the central concept of the piece by delineating between the manifest content *versus* the latent content of dreams. Freud’s dream work elaborates the disconnection between manifest and latent ideas “*into a relatively unified dream-image*” ([8], para. 844). After distancing himself from Freud, both on personal and academic fronts, Jung unequivocally challenges the façade under which the latent content is supposed to hide. 

Initially, Jung [8] aligns with Freud regarding the division of dreams into three classes representing: (1) an unrepressed wish in undisguised form (infantile type); (2) the fulfillment of a repressed wish in disguised form (most dreams belong to this type); and (3) a repressed wish in undisguised form (often accompanied by fear). As for the purpose or function of dreams in general, Jung, in agreement with Freud, articulates succinctly that: dreams are a façade for the preservation of sleep and represent the “guardian of sleep” ([8], para. 866–868). 

Jung pays homage to Freud’s methodology in “The Analysis of Dreams” [10], where he argues that Freud’s method is based on empiricism alone—namely, the common experience that no psychic (or physical) fact is accidental. It must have, then, its train of causes, being always the product of a complicated combination of phenomena; for every existing mental element is resultant of anterior psychic states and ought in theory to be capable of analysis ([10], para. 66). However, after distancing himself from Freud, Jung theorizes a differing view: that “*dreams are often anticipatory and would lose their specific meaning completely on a purely causalistic view*” ([11], para. 312).

In a response article entitled “Morton Prince, ‘The Mechanism and Interpretation of Dreams’: A Critical Review” [12], Jung refutes Prince’s criticism of Freud’s psychoanalytic theory of dream formation and interpretation. From the outset, Jung retains a diplomatic stance supporting Freudian theory. However, Jung gradually becomes ferociously critical, and even condescending, in his subsequent and thorough counter-criticism of Prince’s work. For instance, the “anxiety dreams” reportedly experienced by Prince’s patients, Jung asserts, “*must be regarded from the standpoint of the sexual theory, unless Prince succeeds in proving to us that the sexual theory of anxiety is wrong*” ([12], para. 184). Jung meticulously dissects Prince’s criticism by pointing out the errors and deficiencies in Prince’s interpretations of six dreams of a middle-aged female client who developed a dependency on Prince. At this stage, Jung contends that Freud’s approach scientifically and authentically describes what one *is*. In contrast, Prince’s moralizing of his clinical cases represents a rather pretentious assigning what one *should be*. For Jung, this was a serious flaw in Prince’s approach.

Whereas the aforementioned three articles trumpet and defend Freud, “Association, Dream and Hysterical Symptom” provides an example of Jung applying Freudian theory, especially sexual theory, to rigorous clinical practice. Here, Jung shows in great detail his analytical process, which was in alignment with the Freudian sex-based dream theory of interpretation. In that work, Jung examines nine serial dreams of a 24-year-old inpatient. The work is an intricate and thorough case study, which, due to space limitations, cannot be elaborated any further here. It suffices to say that no direct confirmation came from the patient, and the “dream-analysis and the analysis of the illness as a whole remain[ed] incomplete” ([2], para. 843). Nonetheless, Jung concluded that there was “*something about the brother that goes beyond a sibling relation*”—and that the patient had a “Freudian trauma.” Jung holds that all these dreams indicate “*an intensive sexual complex, wherein the dreams are about nothing but the theme of mating*” [2]. Here, Jung could not be a more faithful proponent of Freudian dream analysis, in addition to psychoanalysis in general. 

## 3. Jung’s Original Contribution—Dream as Compensation

Jung’s original contributions to the interpretation of dreams are multiple, encompassing compensation theory, symbolism, direct image association, the archetypal unconscious, individuation, two-mind confrontation, and the analysis of dreams on both subject and object levels. It is probably safe to say that among Jung’s foremost contributions on dream interpretation, “compensatory theory” is the more important. In some way, the theory becomes the cornerstone of the edifice of Jungian dream interpretation. 

In 1934 Jung wrote: “Every process that goes too far immediately and inevitably calls forth compensation”, and that, “[t]he theory of compensation is a basic law of psychic behavior [...] When we set out to interpret a dream, it is always helpful to ask: What conscious attitude does it compensate ([5], para. 330)?” Thus, my article will focus on this compensatory concept and practice, and proposes that his other contributions formed a constellation around this central compensation theory.

Jung’s remarks on the compensatory nature of dreams are scattered in many articles. Three articles, however, deserve major attention, namely: “General Aspects of Dream Psychology” (*Aspects* hereafter) [4], “The Practical Use of Dream Analysis” (*Practical Use* hereafter) [5], and “On the Nature of Dreams” (*Nature* hereafter) [3]. As the title itself suggests, *Aspects* deals with several important aspects of dreams, and devotes much attention to discussing compensation theory and its clinical applications. *Practical Use* not only designates compensation as “*the basic law of psychic behavior”* and *“one of the best-proven rules of dream interpretation*” ([5], para. 330), but also lays out the details of methodological skills, such as the directed association of images. *Nature*, the shortest of the trio, discusses exclusively the compensatory function of dreams as its true nature. 

Interestingly enough, according to the editors’ footnotes, both *Aspects* and *Nature* had their last editions published in the same year (1948). From the *CW* footnotes, there is barely a way to tell which one of the two was written and published first. The order of their appearance in *CW* might be the only clue for an educated guess. *Aspects* therefore is assumed to precede *Nature*. I rely more on *Nature* than *Aspects*, not only because of the exclusiveness of the topic of the former, but also because of its presumably later date of penmanship and publication.

Despite discussions in *Aspects, Practical Use*, and elsewhere, it is *Nature* that marked the maturity of Jung’s compensation theory. In terms of the general mechanism of dream formation, compensation “*means balancing and comparing different data or points of view so as to produce an adjustment or a rectification*” ([3], para. 545). In *Nature,* the compensation theory is summarized in three possibilities or manifestations: (1) opposites, (2) satisfaction with slight modifications, and (3) parallels or coincidences. Formal definitions of these terms are as follows:

(1) Compensation as opposition to the tendency of the conscious mind if the conscious life situation is “in large degree one-sided.”
(2) Compensation as satisfaction, with slight modification or deviation from the conscious life situation; this type of compensation does not go to extremes and is “fairly near the middle.”
(3) Compensation as emphasizing or coinciding with the conscious attitude if the attitude is the best possible, or “correct” one. This kind of compensation (*i.e*., what is dreamt of coincides with what happens in conscious life) is also known as a parallel compensation [3].

Most of the cases that Jung cites for the compensatory theory allude to dream manifestations as opposites. The following cases are typical for this category. 

(A) Jung was seeing a patient, who was a highly intelligent woman. Jung’s analysis with her dream went well at first, but after a while he got stuck with the interpretation and noticed a shallowness in the dialogue with the analysand. Jung decided to communicate this to the patient. He then had a dream the night before he was to meet with her again. The dream is as follows:

I was walking down a highway through a valley in late-afternoon sunlight. To my right was a steep hill. At its top stood a castle, and on the highest tower there was a woman sitting on a kind of balustrade. In order to see her properly, I had to bend my head far back. I awoke with a crick in the back of my neck. Even in the dream I had recognized the woman as my patient.

([7], p. 133; [13], para. 281)

The interpretation of the dream was immediate and crystal clear to Jung: if in the dream he had to look up at the woman, his analysand, then in waking life Jung had probably been looking down on her both intellectually and morally, as according to Jung, “*dreams are, after all, compensations for the conscious attitude*” ([7], p. 133). Jung shared his dream and interpretation of it with the patient and it produced an immediate positive change in the effect of her treatment thereafter. 

(B) A patient who consciously thinks of himself as an individual with no moral problems dreams of “a drunken tramp willowing in a ditch beside the road” ([6], para. 507). 

In *Nature*, Jung warns that sometimes and in certain cases, such as in the latent psychoses of hereditarily “tainted” individuals, “*compensation may lead to a fatal outcome owing to the preponderance of destructive tendencies”* ([3], para. 547). A brief comparative study is sketched by Caifang J. Zhu [14] outlining the possibility of becoming psychotic due to the integration of the unconscious into consciousness during the process of analytical psychotherapy and the practice of Daoist or Buddhist meditations. 

Turning to compensation types 1 and 2, we may begin with the Daoist sage, ZHUANG Zi (庄子) (alternatively spelled as CHUANG Tzu), who says that one dreams at night what he or she thinks during the day. An antecedent of what Freud called “the day residue” effect, Zhuang Zi seems to favor the ubiquity of parallel dreams, as well as similar (satisfaction-with-slight-modification) type dreams. H. Dieckman [15] wonders if Jung or his analytical psychology overestimates the differences between dreams and waking experiences. G. William Domhoff proposes that “*there is a continuity between dream content and waking thought*” ([16], p. 21). Domhoff goes as far as saying that dreaming consciousness is “*a remarkably faithful replica of waking life”* ([17], p. 9). Later, he softens his tone after summarizing multiple researchers’ findings and views, by concluding that “*dreams are most often reasonable simulations of waking life that contain occasional unusual features in terms of settings, characters, or activities*” ([17], p. 15).

Manifestation 2 of the compensation theory is disproportionately less articulated and much more enigmatic and obscure than manifestation 1. Jung’s available elaborations on manifestation 2 span only a couple of sentences in para. 546 and 568 of *Nature*. This relative diminishment is elucidated further by von Franz in her book, *Dreams*. Here, she surmises that manifestation 2 “*completes what is lacking in those contents of consciousness which are too narrow or are not considered sufficiently valuable (complementary)*” ([18], p. 4). She presents as an example someone who has a “s*uperficially felt sympathy”* for his heterosexual partner in waking consciousness, whilst at night dreaming of a passionate love scenario with the sex partner. In this example, von Franz reasons that the dream “*complements the stronger emotional importance of what has been recognized consciously, an importance which has been overlooked*” ([18], p. 4). Complementation here occurs here as a means of compensation. That is to say, the dreamer brings the suppressed emotion (“superficially felt sympathy”) in waking life to a full play in the dream.

Jung states that manifestation 3, *i.e.*, compensation as confirmation or parallel to a life situation, is actually “rather rare.” Jung does not elaborate why this is so, but simply asserts this as a conviction essentially based on his own experiences ([19], para. 48). Post-Jungians, such as Thayer Greene [20], James A. Hall [21], and Marie-Louise von Franz [17] continue to hold to this “rarity” position (this generally applies to manifestation 2, as well). Hall tries to explain it away in three ways:

(1) The report of parallel dreams—what Hall calls “dreams of reality-as-it-is”—is often erroneous because symbolic elements, when carefully inquired into, often turn out to be “*significantly different from the reality of the dreamer’s waking life*” ([21], p. 90).
(2) Such dreams may not be “truly” dreams. That is, such dreams may occur at certain levels of consciousness during sleep, and such dreams resemble waking consciousness (such as in meditative states) ([21], p. 90). (We should be aware that the result of experimental study on lucid dreaming was presumably not published at the time James Hall wrote this.)
(3) If it does happen, then “the unconscious intends the waking situation to be viewed as if it were a dream” ([21], p. 91).

Few further references are available pertaining to Jung’s explanation as to why parallel dreams are purportedly so rare. My hypothesis is that it might have to do with the development of personality (self-centered in Jungian sense *vs.* ego-centered), the era (ancient/pre-modern times *vs.* modern), and cultural milieu (dialectical/dynamic/integrative culture *vs.* dichotomist/rational/linear thinking) [22]. It would be plausible to assume that people of the latter category of each dyad are more egocentric or less balanced than people of the former category, so that the latter (*i.e*., modern Westerners in general) manifest greater unconscious compensation for their conscious behavior. For Jungian and non-Jungian dream workers alike, perhaps it is time to explore this hypothesis with experimental studies in existent sleep and dream labs. 

## 4. Jung’s Final Stand on Dreams: Relative and Fluid

After approximately 60 years of work on dream interpretations, Jung concluded in his “Symbols and the Interpretation of Dreams” that:

There is no rule, let alone a law, of dream interpretation, although it does look as if the general purpose of dream is compensation. At least, compensation can be said to be the most promising and most fertile hypothesis.

([6], para. 507)

Jung was considerably influenced by the Eastern wisdom traditions. One wonders how much of Jung’s final position on dreams is a reflection of the dialectical, dynamic, and fluid philosophies of the East. In the same vein, we can ask how the Daoist philosophy of Yin-Yang regulation and reversion as the motion of Dao/Tao, and the concept of “enantiodromia” [13] Jung attributed to Heraclitus possibly influenced the shaping of Jung’s compensatory theory. Exploration along this line deserves a separate paper to investigate such a hypothesis.

The essay *Symbols* is quite likely the very last paper, or at least one of the last papers, Jung wrote himself because it was written in 1961, the year he died. According to the editorial footnotes of *Symbols*, Jung composed the essay in English without a title. It was originally written as a contribution, together with four of his colleagues, towards a symposium called *Man and His Symbols*, which contributed to popularizing Jung’s ideas. It is not known via the notes, however, whether Jung actually made it to the symposium or not. What is clear is that Jung’s essay was, under the supervision of John Freeman and Marie-Louise von Franz, extensively re-worked and re-written with Jung’s agreement, presumably to meet the purpose of a popular presentation. The version collected in *CW 18* that I am referring to, to our delight, is Jung’s original text. It is revised, however, by R. F. C. Hull, the principal translator of the bulky *CW.* The original organizational arrangement, except for some minor transpositions, has been kept intact. The title and sub-titles of four sections were all added post-production. 

The assumption in the authenticity of Jung’s definitive stand on dreams in *Symbols* finds support from a larger contextual study. There are a number of places in section four of *Symbols* (“The Problems of Types in Dream Interpretation”) that re-state in different expressions this definitive stand on the nature of dreams and interpretation. For example, paragraph 495 [6] reads, “*The process of interpretation consists in the confrontation of two minds, the analyst’s and the analysand’s, and not in the application of a preconceived theory.*” As a dream interpretation process, or the systematic analysis of dreams, requires a confrontation of two minds, Jung believed it would make a great difference if their types of attitude were the same. To Jung, himself an introvert, the extroverted Freud belittled introverted patients as morbidly engrossed in themselves ([6], paras. 498–499). The dynamics of the analyst and the analysand, rather than any preconceived theory, thus determine much of the process of dream interpretation. Alongside this, Jung reaffirms:

If you want to understand another person’s dream, you have to sacrifice your own predilections and suppress your prejudices [...] if you don’t make the effort to criticize your own standpoint and to admit its relativity, you will get neither the right information about, nor sufficient insight into, your analysand’s mind […] one has to remind oneself again and again that in therapy it is more important for the patient to understand than for the analyst’s theoretical expectations to be satisfied.

([6], para. 505)

Unmistakably, here Jung was pointing to the relativity of truth pertaining to any theory, including his own on dreams. By this, he was also pioneering humanistic psychology and a client-centered approach to psychotherapy [23]. 

Echoing no general rule (nor the law of dream interpretations), Jung reflected that 60 years of clinical practice had taught him that there was “*no therapeutic technique or doctrine that is generally applicable*” ([6], para. 515). Rather, he had to “*regard each case as a new experience, for which, first of all, I have to seek the individual approach*’ ([6], para. 518). According to Jung, two individuals can have almost identical dreams, but if one is young and the other old, their perception and experience of the dream-scope may be markedly different. If anything, Jung proposed that maintaining the rapport with the patient and following his or her inclination, supported by his or her own dream, is optimal. 

Jung’s final comments on the relativity of compensation as dream interpretation theory was heralded in articles published prior to *Symbols*. In *Aspects* alone, the formulation of such insights were emerging and expressed as follows:

It is therefore not easy to lay down any special rules for the type of dream-compensation. Its character is always closely bound with the whole nature of the individual. The possibilities of compensation are without number and inexhaustible, though with increasing experience certain basic features gradually crystallize out.

([4], para. 490)

A dream, like every element in the psychic structure, is a resultant of the total psyche … so the dream cannot be explained by this or that element in it, however beguilingly simple such an explanation may appear to be [...] In order to do anything like justice to dreams, we need an interpretive equipment that must be laboriously fitted together from all branches of the humane sciences.

([4], para. 527)

This definitive stand also finds antecedent voices in the essay *Nature*, where Jung states that the only justifiable interpretations of dreams are reached through a painstaking examination of the context. In doing so, the interpreter, however experienced he or she may be, is always obliged “*to admit one’s ignorance and, renouncing all preconceived ideas, to prepare for something entirely unexpected*” ([3], para. 543). Renouncing all preconceived ideas necessarily includes that of compensation theory. Epistemologically or cognitively, this is reminiscent of Freud’s analytic technique of “*equally suspended attention*”. Likewise, phenomenologists contemporaneous with Jung, such as Edmund Husserl [24] and Maurice Merleau-Ponty, employed differing nomenclature to express a similar message: bracket, eliminate or suspend one’s presuppositions to let things manifest themselves as they really are [25]. In Chan/Zen and Vipassana meditations, practitioners look inward at the thoughts, emotions, and memories whose constant flux constitute mental phenomena—the “original face” or the original nature of the mind. Heidegger [26] and Gadamer [27] assert that only after one has arrived at the pre-conceptual, pre-suppositional, and pre-judgmental state of mind can one’s hermeneutics be justified as a “*fusion of horizons.*”

In the last paragraph of *Nature*, Jung wrote that, in the study of dream psychology, we cannot boast that we have possessed:

a generally satisfying theory or explanation of this complicated phenomenon. We still know far too little about the nature of unconscious psyche for that […] For the purpose of research is not to imagine that one possesses the theory which alone is right, but, doubting all theories, to approach gradually nearer to the truth.

([3], para. 569)

If the doubting of theory allows us to come closer to the truth*,* then it is easy for us to understand why Jung hardly expresses any sense of positive construction in *MDR*. In retrospect, in the last chapter of *MDR*, Jung writes, “*There is nothing I am quite sure about. I have no definite convictions—not about anything, really*” ([7], p. 358). A man of advanced age, he ended up quoting the Chinese Daoist sage Laozi (Lao Tzu 老子), “*All is clear, I alone am clouded*” ([7], p. 359). Here, Jung appears deconstructive and post-modern, which is ironically pre-modern in the East-Asian wisdom tradition.

In fact such thoughts of deconstruction and openness can be traced further back in Jung’s works. For example, in “The Aim of Psychotherapy,” Jung could not have made it clearer when he wrote, “*I have no theory about dreams; I do not know how dreams arise. I am altogether in doubt as to whether my way of handling dreams even deserves the name of ‘method*” ([11], para. 86). No law, no rule, not even a method is assured. On the other hand, Jung continued: “*if we meditate on a dream sufficiently long and thoroughly, if we carry it around with us and turn it over and over, something almost always comes of it*” ([11], para. 86). That is, there are techniques of interpretation (e.g., the directed association of dream images and symbols), although there is no general law or principle. In his chapter, “C.G. Jung’s theory of dreams,” Greene cited the above referenced para. 86. However, he did not see it as a final stand from a developmental viewpoint.

Readers may ask: since Jung’s open stand is traced back to the early 1930s, how does my proposition make sense that it is within the last several years of his life that Jung’s definitive theoretical stand was clearly formed? In answer to this, Jung [5], as in *Practical Uses* for instance, was much more enigmatic or ambivalent on the relation between compensatory theory as a law or rule, as opposed to it being a thoroughly open stand. Through a maturation process, however, culminating in *Symbols,* he unequivocally declares his definitive open stand: compensatory theory is just a hypothesis. 

A physician and contemporary of William James and Edmund Husserl, Jung was apparently more pragmatic [28] and phenomenological than scientific, as he sometimes controversially claimed himself to be [29]. As long as his interpretation means “*something to the patient and sets his life in motion again*,” Jung said he was wholly content with himself, leaving explanations as to “why it works” for his “spare time” in the form of “scientific hobby” ([11], para. 86).

## 5. Challenges and Confirmations from Cognitive and Neuroscientific Dream Theories

In *The Multiplicity of Dreams*, Harry T. Hunt [30] constructed a cognitive psychological interpretation of dreaming that distinguished dream psychology mediated by left hemispheric brain structures, which emphasize functions of language and memory, from dream psychologies mediated more by right hemispheric brain structures, which are associated with the processing of imagistic and organismic–holistic cognition. The former camp is represented by Sigmund Freud, David Foulke, and Allan Hobson, and the latter, by Jung and James Hillman [31], among others. For Hunt, the “polysemy and multiplicity” of dreams “deny any single fixed interpretative meaning or underlying structure” ([30], p. 208). This echoes Jung’s final stand on dream interpretations. Though Hunt classified Hobson in the camp that analyzed left-brained dream psychology, Hobson noted the complexity of the issue by quoting his colleague Bob Stickgold: “*Freud was 50 percent right and 100 percent wrong*” ([32], p.148). Moreover, Hobson appears to be much more affirming and appreciative of Jungian contributions, as opposed to wholly critical. In relation to Jung’s dream theory, Hobson and Hunt are close on one thing at least: both barely mention compensation as the core substance of Jung’s dream theory. 

Hunt goes to great lengths to justify and prioritize the reflexive presentational process of imagistic symbolic cognition over the verbal-representational cognition of labeling and thinking in language. To Jungians, this confirms the centrality of dream images, especially in archetypal or titanic dreams. Jean Knox [29,33], however, argues that Jungians have reified the unconscious structures, such as archetypes and the Self. The image schemas or archetypes that she challenges are “*an early developmental conceptual achievement rather than being an inherited innate psychic component*” ([32], p. 316). Moreover, although the concept of an archetype is “the earliest true concept,” it is developed “*after motor abstraction in the abstraction/de-coupling process*” has been completed ([33], p. 316). Knox [33] certainly has to explain more concerning how the activation of mirror neurons’ intentionality (however she defines intentionality) leads to the emergence of concept formation, not to mention the emergence of social or interpersonal interaction. 

Hunt has outlined his contemplations on the proposition that so-called “reality” is an illusion or dream, stemming from cross-cultural perspectives. He appreciates the Eastern Wisdom Traditions where “*[t]he adept is freed when long-term meditative practice renders the experience of waking reality as ephemeral as a dream*” ([30], p. 217). However, he complains that since the positivism and rationalism of Descartes in the West, viewing life as a dream has been considered a “*metaphysical horror to be refuted at all costs”* ([30], p. 217). Like many others, here Hunt risks making a contrast using an unbalanced method/approach. At best, he is contrasting the selected few hundred years of the Western intellectual tradition with thousands of years of the Eastern Wisdom Traditions, within which various competing schools existed.

Hobson [28,31] takes an empirical position regarding dream science that boils down to the activation–synthesis theory. We dream, according to Hobson, because our brains, while we are in sleep, randomly and reflexively activate themselves in the brain stem, whereby these random activations are synthesized in the frontal lobes. In relation to Jung’s approach to dreams, Hobson’s major challenge is to downplay the meaning of dreams to the point where dream contents are no longer worthy of analyzing; such downplaying is premised on the proposition that dreams are basically biochemical activities of the brain that can be just as apparently “meaningless” as delirium or psychosis. Thus, under this framework, instead of asking what the dream possibly means, Hobson claims to have shifted the paradigm by asking what the mental (perceptual, cognitive and emotional) characteristics of dreaming are. 

Still, in our case, Hobson deserves our attention for at least two major reasons. First, Hobson does not pinpoint Jung’s dream theory to compensation. Instead, Hobson points out that “*Jung’s dream theory emphasizes transparency and creativity, in contrast to Freud’s emphasis on obscurity and psychopathology*” ([28], p. 65). What does Hobson possibly mean by saying Jung’s dream theory emphasizes “creativity”? Is he confirming the openness, fluidity, and relativity that characterizes the third phase of Jung’s dream theory developmental trajectory? Probably yes. Cognitively speaking, creativity does presuppose openness or freedom of the habitual mindset. By “transparency,” Hobson was likely referring to the directedness of Jungian dream symbolism, which directly expresses universal human concerns from the unconscious, and especially those concerns that emerge from the collective unconscious. Hobson, Greene, Hall, Samuel, and Whitmont [34], among others, all appreciate Jung’s directness over Freud’s disguise–censorship theory. Echoing Jung, Hobson does not believe in the disguise function of the latent layer of dream contents. Hobson tends to hold that “*dreams reveal rather than conceal emotion and instinct,*” and therefore he concludes that “*disguise–censorship is not only unnecessary but misleading. In fact, it is downright erroneous*” ([31], p. 151).

The second point of Hobson’s views that merits our attention pertains to Jung’s ambiguous but pragmatic stand in relation to science. Hobson, aligning Jung with William James for their shared interests in psycho-spirituality, writes:

Pragmatic and experimental, they refused to accept any hypothesis that was not scientifically justified. Meanwhile, phenomena that could not be so explained were not denied existential status, ex cathedra. Thus, the apparent religiosity of James and Jung can obscure their fundamental scientific rigor.

([28], pp. 67–68)

Hobson’s comment has a two-fold meaning. First, it continues to support our reasoning above, that Jung saw his practice and theory of dreams (if there is any so-called theory he subscribed to) as having an open, fluid, and relative status. Such a status accounts for creativity in a two-mind therapeutic dynamic that is constantly changing intra-psychically and interpersonally, and involves subject-and-object levels of interpretation. Second, it is more congenial to see Jung as a pragmatist (*i.e*., a pragmatic analyst) rather than as a scientist; Jung kept claiming to be scientific at times and “ruefully” acknowledged deviating far from scientific norms [29]. Jung’s work is an exemplar of the human science with which phenomenologists, existentialists, hermeneutics, and humanistic and transpersonal psychologists have been defending themselves with since the onset of the post-modern era; such individuals struggle against the excess and dominance of the Western natural sciences [26,27,35]).

Hobson claims that the most significant conclusion of his new dream theory is that it can predict that brain activation of a given chemical and regional type will always produce hallusinosis, hyperassociativity, hyperemotionality, false beliefs, and other cognitive errors. This is as far as scientific prediction can now go with dreams, but it is far enough to put the formal psychological analysis of dreams out of the reach of content analysis.

([32], p. 158)

In his theory of dream formation, Hobson goes to great lengths to prove with experimental data (predominantly via empirical data with animals) that dreams are chemically mediated and enhanced within the brain. For example, he states, “*We can safely conclude that REM sleep dreaming is mediated by acetylcholine when noradrenaline and serotonin are at very low levels*” ([32], p. 69). The excitability level of cholinergic neurons, however, is subject to “a wide variety of genetic and experimental factors that contribute to long-and short-term differences in sleep, which are correlated with normal development, learning, and memory, and even mood and temperament” ([32], p. 70). Having reduced the concept of the “mind” to a self-activating brain, and the process of dreaming to cholinergic neuron stimulation, Hobson nonetheless takes a step back by using the term “brain-mind” and admitting that the self-activating brain’s “*capacity for subjectivity remains to be explained*” ([31], p. 64). 

Hobson attempts a defense against reductionism, or at least against a rigid reductionism, by reasoning that why dreams are so hyperassociative, instinctive, emotional, and perceptually intense, is because “*the brain regions supporting these functions are more active*” ([32], p. 113). In the same light, the reason we cannot keep track of time, place, and person, nor think and judge rationally and critically regarding our dreaming is simply because these brain regions were functionally less actively at the time. Hobson asserts; “*This is the true meaning of reductionism*” ([32], p. 113), implying his version of reductionism allows for leeway, ranging from the single neuronal level (acetylcholine) to regional levels of the brain. Alternatively, he avoids rigid reductionism via the synergic component of his activation–synthesis theory. Hobson proposes that when the brain stem randomly self-activates in sleep, the forebrain synthesizes the random activation into something like waking experience. While Hobson mitigates his reductionism in this way, Knox arguably sidesteps a reductionist snare with recourse to attachment theory. Knox concludes that the clinical phenomena indicating that many patients are stuck at the teleological or any other of the six levels of self-agency “*may be seen as a failure to develop the abstract generalized image schema of self-agency that can integrate all the specific levels or frames of reference of self-agency*” ([33], p. 320). Image is one of the key words used in Jung’s interpretation of dreams.

Hobson’s activation–synthesis theory of dream formation has been challenged by Mark Solms [17] since 1997. Based on his neurophysiological research on patients’ reports, Solms finds that (1) dreaming originates in the ventral tegmental area of the midbrain, a few centimeters from Hobson’s pons area of the brain stem that generates REM sleep; (2) many dreams can happen in non-REM sleep (a phenomenon that Hobson had attempted to explain away); and (3) it is a high level of dopamine, rather than acetylcholine, which is significant in modulating and enhancing dreaming process [17,36]. 

Domhoff believes that the empirical and highly technical differences between Hobson and Solms are resolvable through research findings. Domhoff thus challenges both men for being “hard-nosed” scientists and refutes much of their neuropsychological speculation and theory “*because the main findings on dream content are at odds with them*” ([17], p. 17). Instead of relying on neurophysiology merely for formal analysis, Domhoff calls for “a new neurocognitive theory of dreams” ([17], p. 18) that blends studies pertaining to features of dreaming and waking cognitions with dream content using the extant neuroscientific technologies and findings. This seems to be a sensible new approach to the study of both dream formation and interpretation because it integrates the value of the dream content that Jung emphasized with cognitive and neuroscientific findings.

## 6. Conclusions

The full landscape of Jung’s dream theory trajectory should contain three periods, despite the fact that there is some overlap of time between the phases. To single out one phase at the cost of the others is to provide only a partial picture. The developmental delineation of this paper features a solid contextual and textual exegesis that uses primary sources (although most of them are English translations). Hypotheses have been presented as to why unconscious compensation, as a parallel and/or satisfaction with slight modification, is so rare. References to the possible influences from Eastern thought, as well as from phenomenology and pragmatism, are also briefly mentioned. Cognitive and neuroscientific theories of dream formation both challenge and confirm the aspects of Jungian theory that concern dreams and interpretation. Our understanding on dreaming requires greater integrative applications. Cognitive and neuroscientific approaches can and should be combined fruitfully with the Jungian approach. 
